# Three-dimensional distribution of CT attenuation in the lumbar spine pedicle wall

**DOI:** 10.1038/s41598-020-80676-5

**Published:** 2021-01-18

**Authors:** Tomoyo Y. Irie, Tohru Irie, Alejandro A. Espinoza Orías, Kazuyuki Segami, Norimasa Iwasaki, Howard S. An, Nozomu Inoue

**Affiliations:** 1 Department of Orthopedic Surgery, Rush University Medical Center, Chicago, 60612 USA; 2 Department of Orthopaedic Surgery, Faculty of Medicine and Graduate School of Medicine, Hokkaido University, Sapporo, 060-8638 Japan

**Keywords:** Bone, Translational research

## Abstract

This study investigated in vivo the three-dimensional distribution of CT attenuation in the lumbar spine pedicle wall measured in Hounsfield Unit (HU). Seventy-five volunteers underwent clinical lumbar spine CT scans. Data was analyzed with custom-written software to determine the regional variation in pedicle wall attenuation values. A cylindrical coordinate system oriented along the pedicle’s long axis was used to calculate the pedicular wall attenuation distribution three-dimensionally and the highest attenuation value was identified. The pedicular cross-section was divided into four quadrants: lateral, medial, cranial, and caudal. The mean HU value for each quadrant was calculated for all lumbar spine levels (L1–5). The pedicle wall attenuation was analyzed by gender, age, spinal levels and anatomical quadrant. The mean HU values of the pedicle wall at L1 and L5 were significantly lower than the values between L2–4 in both genders and in both age groups. Furthermore, the medial quadrant showed higher HU values than the lateral quadrant at all levels and the caudal quadrant showed higher HU values at L1–3 and lower HU values at L4–5 than the cranial quadrant. These findings may explain why there is a higher incidence of pedicle screw breach in the pedicle lateral wall.

## Introduction

Pedicle screw fixation has become a gold-standard method of stabilization for a variety of spinal disorders. However, fixation-screw loosening or failure may occur, particularly in osteoporotic patients^1–3^. The new technique of cortical bone trajectory screw fixation has been proposed to increase the fixation strength in osteoporotic spines, by increasing the contact surface between the screw and pedicle cortical bone, which is stronger than the trabecular bone and less influenced by osteoporotic changes^4–6^.

The pedicle is a tubular bony structure with osteons orienting mainly in a longitudinal direction along the pedicle length, similar to long bones^7^. Unlike long bone diaphysis, the pedicle has a trabecular internal structure. Previous studies on pedicle screw pullout strength have mainly focused on the trabecular computed tomography (CT) attenuation values expressed in Hounsfield Units (HU) values in the vertebral body and the pedicle^8,9^. Recent studies using advanced imaging techniques indicated that the local HU values of the trabecular bone surrounding the pedicle screw was a reliable predictor of the pedicle screw pullout strength^10,11^. Although no cortical purchase of the lumbar pedicle screw has been reported in a laboratory setting using cadaveric spines, some degree of misalignment can occur during pedicle screw insertion during surgery, which causes redirection of the pedicle screw and breach of the pedicle wall^12–14^. It has been reported that cortical purchase of the pedicle screw provides higher fixation strength both with traditional pedicle fixation^15,16^ and cortical bone trajectory fixation^4,17,18^.

The pedicular cortex structural properties have been studied mainly with regards to cortical thickness^19,20^. Regional variation of the pedicle isthmus thickness has been previously investigated and a thicker medial cortex compared with the lateral cortex was reported^7,21–23^. Because bone quality of the cortex is important especially for the cortical bone trajectory screw fixation, recent research on cortical bone trajectory aims to establish noninvasive methods to predict bone quality along the cortical bone trajectory screw pathway^24,25^. Bone mineral density has been reported as a reliable predictor of bone material properties, measurable using clinical imaging modalities^6,26,27^. However, to the best of our knowledge, there is no study in the literature describing the three-dimensional (3D) distribution CT attenuation values through the lumbar spine pedicle wall using clinical computed tomography (CT) in vivo. Therefore, the objectives of this study were to investigate the 3D HU values distribution in the lumbar spine pedicle wall in vivo using clinical CT and to analyze variations of the pedicle wall attenuation values by gender, age, spinal level and anatomical site.

## Materials and methods

### Subjects

A total of 84 volunteers participated in this IRB-approved study (Rush University Medical Center Institutional Review Board, No. 00042801) including all subjects signing an approved informed consent form. All research was carried out in compliance with relevant guidelines and regulations. From this initial recruitment group, nine of them were excluded for the following reasons: one volunteer had spondylolisthesis, seven volunteers had transitional lumbosacral vertebrae and the last one had a combination of spondylolisthesis and transitional lumbosacral vertebrae. In consequence, a total of 75 subjects were used for the analyses.

### Imaging

Each subject underwent CT imaging of the lumbar spine (L1-S1, Volume Zoom, Siemens, Malvern, PA, tube voltage: 120 kV, tube current: 100 mA, field of view: approximately 200 mm, image matrix: 512 × 512, slice increment: 1.0 mm, slice thickness: 1.0 mm, no spacing) in a supine position. Axial slice raw image data were exported in the DICOM format.

### Measurement of attenuation in the vertebral body

Attenuation in the vertebral body was measured using the software ImageJ (US National Institutes of Health, Bethesda, MD) to evaluate osteoporotic changes of the vertebral bodies. For each of vertebral bodies the largest possible elliptical ROI was drawn for the vertebral body HU measurement^28^. These ROIs were acquired on all the axial CT slices of the L1–L5 lumbar levels. Osseous abnormalities and voids such as vascular channels were excluded from the ROIs.

### Determination of local density distribution in 3D space adjacent to the pedicle axis measured in Hounsfield Units (HU)

**Figure 1:**
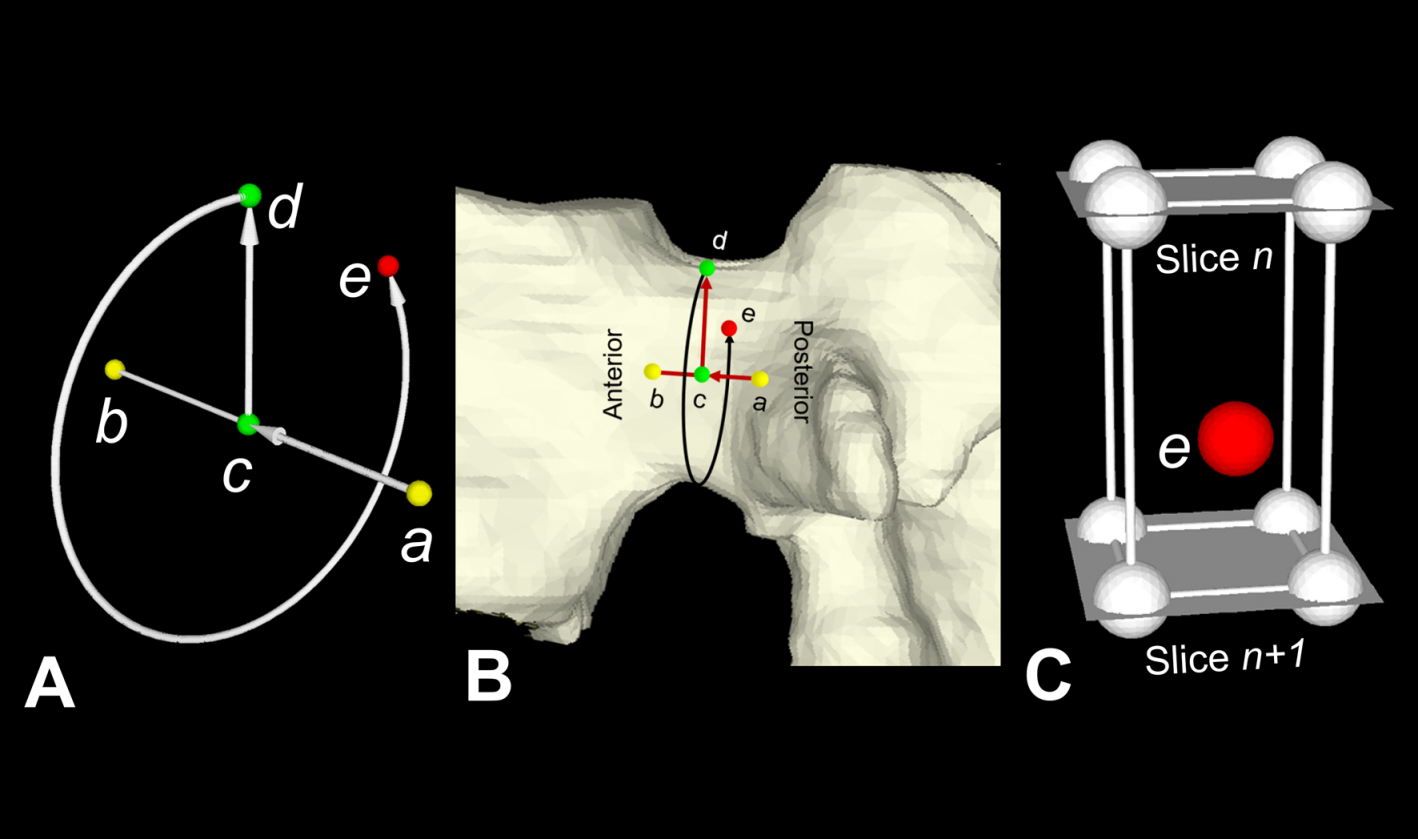
Determination of attenuation in Hounsfield Units (HU) at an arbitrary point in space using a cylindrical coordinate system with its longitudinal axis oriented along the pedicle’s long axis. (**A,B**) An arbitrary point ***c*** moves along a temporary pedicle axis connecting center points of the posterior and anterior pedicle ends (points ***a*** and ***b,*** respectively). Vector $\overrightarrow {{{\varvec{cd}}}}$ is perpendicular to vector $\overrightarrow {{{\varvec{ab}}}}$. The point ***d*** revolves around the pedicle axis and a point ***e*** is defined in the cylindrical coordinate system. (**C**) The HU value of the point ***e*** (red sphere) is calculated from HU values at 8 adjacent points (white spheres) in the 2 adjacent CT slices (slice *n* and slice *n* + 1) using a trilinear interpolation algorithm.

**Figure 2:**
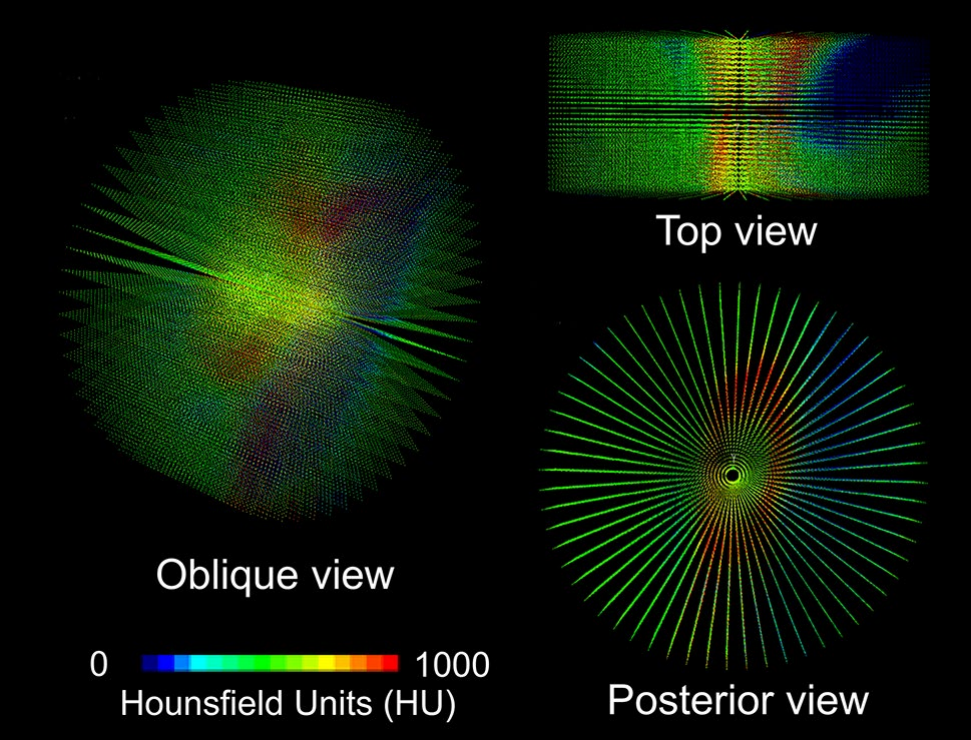
3D attenuation distribution in a cylindrical space centered about the pedicle axis obtained directly from a CT DICOM dataset. The 3D geometry of the pedicle cortex can be inferred from the high HU values with reddish color hues.

**Figure 3:**
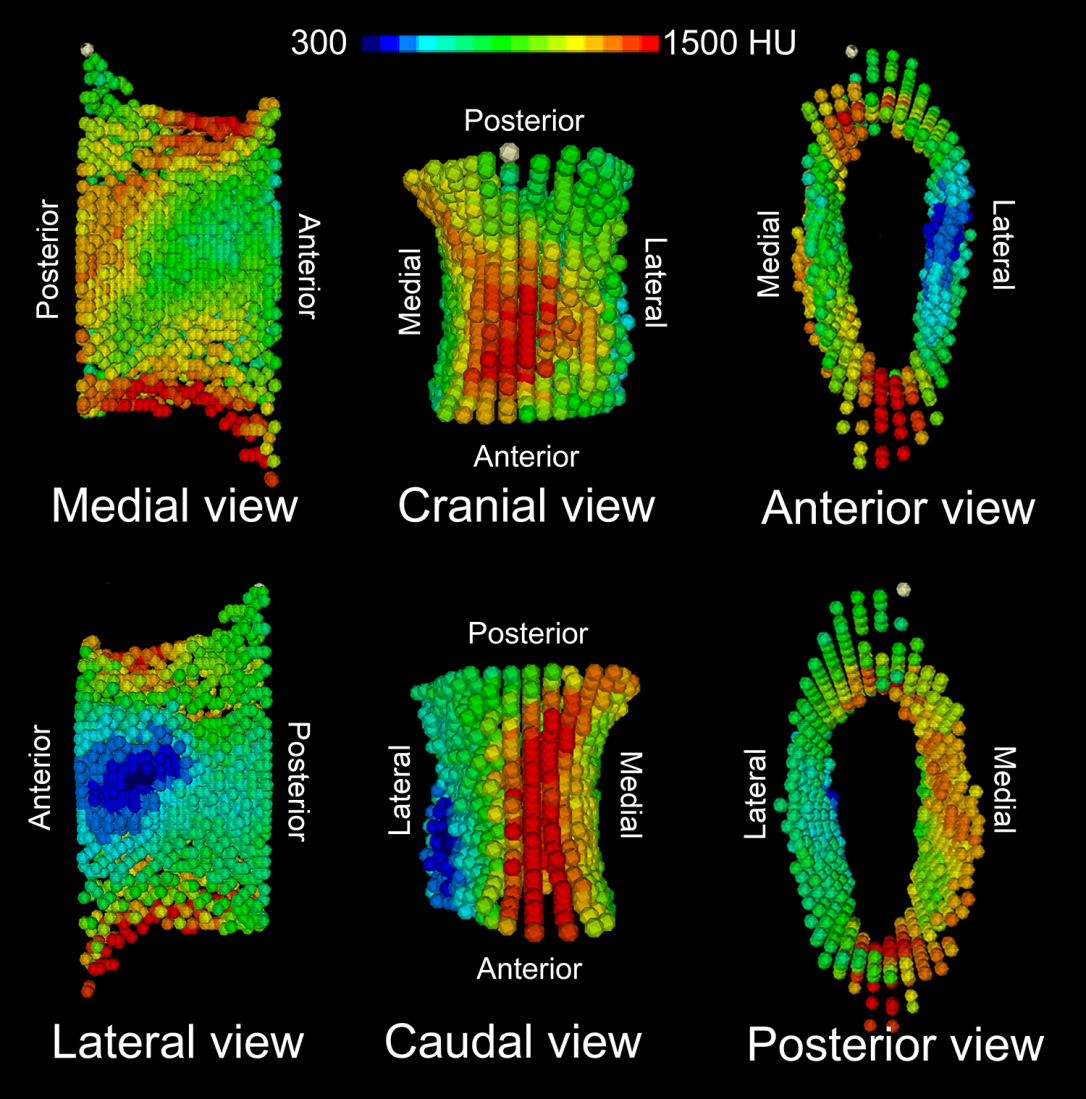
Innovative visualization resulting from the HU values distribution analysis. The pedicle geometry is displayed by the 3D distribution of points with the highest HU values in each discrete radial direction shown in the cylindrical coordinate system.

To obtain the 3D distribution of HU values in the space adjacent to the pedicle axis, a cylindrical discrete coordinate system centered along the pedicle long axis was defined as follows: *Approximate* center points in both the posterior (point ***a***) and anterior (point ***b***) ends of each pedicle were manually determined in the DICOM image(s) of the pedicle level using commercially available software (Mimics R21, Materialise Corp., Leuven, Belgium) (Fig. 1A,B). Point ***c*** was set on line $\overline{{{\varvec{ab}}}}$ and moved from ***a*** to ***b*** with in increments of 1/30 of the length of line $\overline{{{\varvec{ab}}}}$. This enabled the definition of vector $\overrightarrow {{{\varvec{cd}}}}$ in a cranial direction perpendicular to vector $\overrightarrow {{{\varvec{ab}}}}$. Point ***d*** revolved 360° in 6° increments on a plane perpendicular to $\overline{{{\varvec{ac}}}}$ and a probe point ***e*** was determined. The magnitude of vector $\overrightarrow {{{\varvec{cd}}}}$ was increased from 0.5 mm to 13.0 mm in 0.25 mm increments. The HU value at each point ***e*** was calculated by a trilinear interpolation algorithm using HU values at 8 adjacent pixels in 2 adjacent axial CT slices (Fig. 1C)^29^. A total of 90,000 HU datapoints were obtained within the cylindrical range of interest (ROI) shown in Fig. 2. The highest HU value in each discrete radial direction in the discrete cylindrical coordinate system was determined and a total of 1,800 HU datapoints per pedicle were recorded. The discrete cylindrical coordinates of the point at which the highest HU value occurred were recorded. A 3D pedicle model was assembled using the 1800 datapoints to represent the spatial variation in attenuation by anatomical quadrant (Fig. 3).

### Determination of topographic quadrants

Four topographic quadrants: (a) cranial, (b) medial, (c) caudal, and (d) lateral were determined in 3D space, as described next. Since cross-sectional axes decline from the anatomical axes of each vertebra^30^, the longest axis at the isthmus was calculated and defined as the craniocaudal axis for each pedicle. The four topographic quadrants were defined by 90° intervals as follows: cranial: ± 45°; medial: 45°–135°; caudal: 135°–225°; and lateral: 225°–315° from the cranial axis of each pedicle.

### Statistical analyses

Right and left pedicles in the same vertebral level were compared via a paired Student’s *t*-test. A gender effect was analyzed by an unpaired Student’s *t*-test. Age was stratified into two groups: 20 s/30 s and 40 s/50 s. Parameters from these cohorts were then compared via an unpaired Student’s *t*-test. Regional and level effects of the pedicle cortex were analyzed using repeated-measures ANOVA with Tukey’s post hoc test to probe for statistical differences. Correlations between the mean pedicle wall HU values and the vertebral body HU values at each spinal level were analyzed via Pearson’s correlation coefficients. Results were presented as mean ± SD. Significance was set at *p* < 0.05.

## Results

In total, n = 750 lumbar pedicles (L1–5) from 75 volunteers (39 males and 36 females; mean age, 39.3 ± 9.6 years old [range 23–59 years old], mean weight, 74.9 ± 16.9 kg [range 44.5—129.3 kg], mean height, 164.3 ± 12.3 cm [range 133.4—186.1 cm] and mean body mass index, 27.9 ± 7.1 kg/m^2^ [range 18.9—53.5]) were included in this study (Table 1). The mean vertebral attenuation values were 209.3 ± 52.0 HU, 210.4 ± 53.9 HU, 198.9 ± 64.6 HU, 203.0 ± 75.9 HU and 204.3 ± 73.1 HU at L1, L2, L3, L4 and L5, respectively (Table 2). There were no significant differences between levels in total, gender and age groups. Two subjects (one was 45 years old male; one was 23 years old female) had the vertebral attenuation values at L1 less than 110 HU, which was proposed as a cut-off yielding high specificity for osteoporosis by Pickhardt et al^31^*.*

**Table 1:** Subject demographics.

	Male	Female	Total
**Subject (N)**	39	36	75
20 s	4	8	12
30 s	18	12	30
40 s	11	10	21
50 s	6	6	12
**Age (years)**			
Mean ± SD	39.2 ± 8.7	39.3 ± 10.5	39.3 ± 9.6
Range	25–58	23–59	23–59
**Height (cm)**			
Mean ± SD	168.3 ± 14.0	159.5 ± 7.6	164.3 ± 12.3
Range	133.4–186.1	142.2–175.3	133.4–186.1
**Weight (kg)**			
Mean ± SD	81.6 ± 12.5	67.6 ± 18.2	74.9 ± 16.9
Range	67.6–129.3	44.5–107.3	44.5–129.3
**Body mass index (kg/m**^**2**^**)**			
Mean ± SD	29.5 ± 7.0	26.3 ± 7.0	27.9 ± 7.1
Range	23.2–53.5	18.9–43.3	18.9–53.5

**Table 2:** Comparison of vertebral body HU values by gender and age group.

Gender and Age group	L1	L2	L3	L4	L5
Total (n = 75)	209.3 ± 52.0	210.4 ± 53.9	198.9 ± 64.6	203.0 ± 75.9	204.3 ± 73.1
Male (n = 39)	203.5 ± 43.2	211.7 ± 46.6	199.4 ± 57.0	195.0 ± 61.2	203.2 ± 62.8
Female (n = 36)	215.5 ± 60.1	208.9 ± 61.6	198.4 ± 72.7	211.6 ± 89.3	205.6 ± 83.7
20’s + 30’s (n = 42)	218.0 ± 55.4	213.6 ± 53.1	210.6 ± 71.5	218.0 ± 80.3	206.1 ± 73.0
40’s + 50’s (n = 33)	198.2 ± 45.8	206.3 ± 55.6	184.1 ± 51.9	183.8 ± 66.3	202.1 ± 74.2

There were no significant differences between levels in total, gender and age group. There were no significant differences between males and females, and between 20’s + 30’s and 40’s + 50’s at all levels.

**Figure 4:**
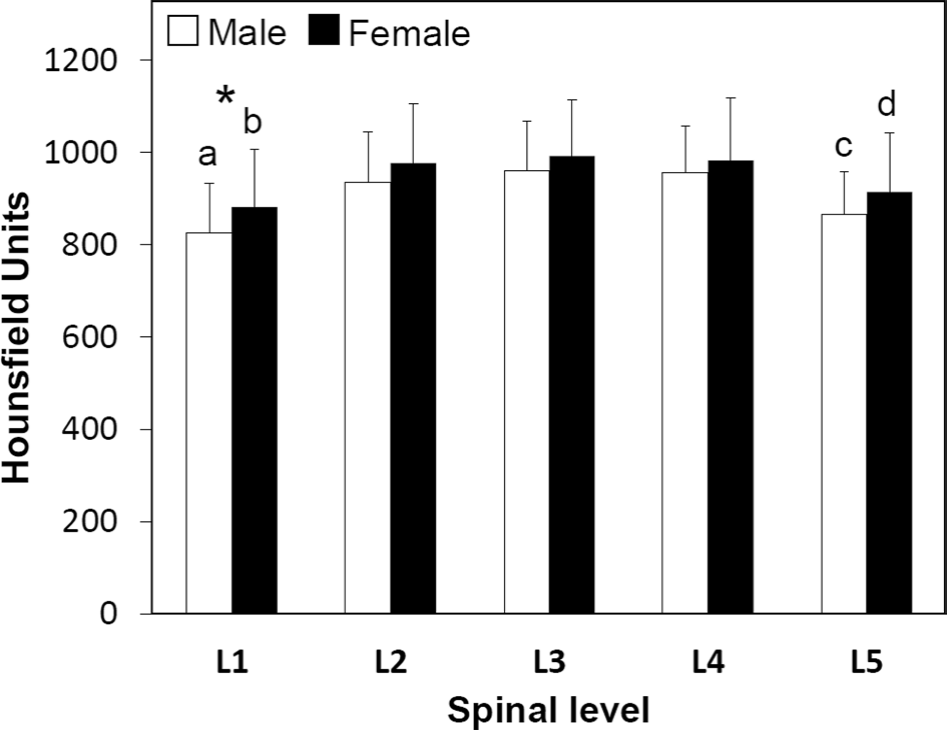
Attenuation split by spinal level and gender. *p < 0.05 between males and females, (**a**) *p* < 0.0001 compared with L2, L3 and L4 in males, (**b**) *p* < 0.0001 compared with L2, L3 and L4 in females, (**c**) compared with L2 (*p* < 0.0006), L3 (*p* < 0.0001) and L4 (*p* < 0.0001) in males, (**d**) compared with L2 (*p* < 0.05), L3 (*p* < 0.003) and L4 (*p* < 0.003) in females.

**Figure 5:**
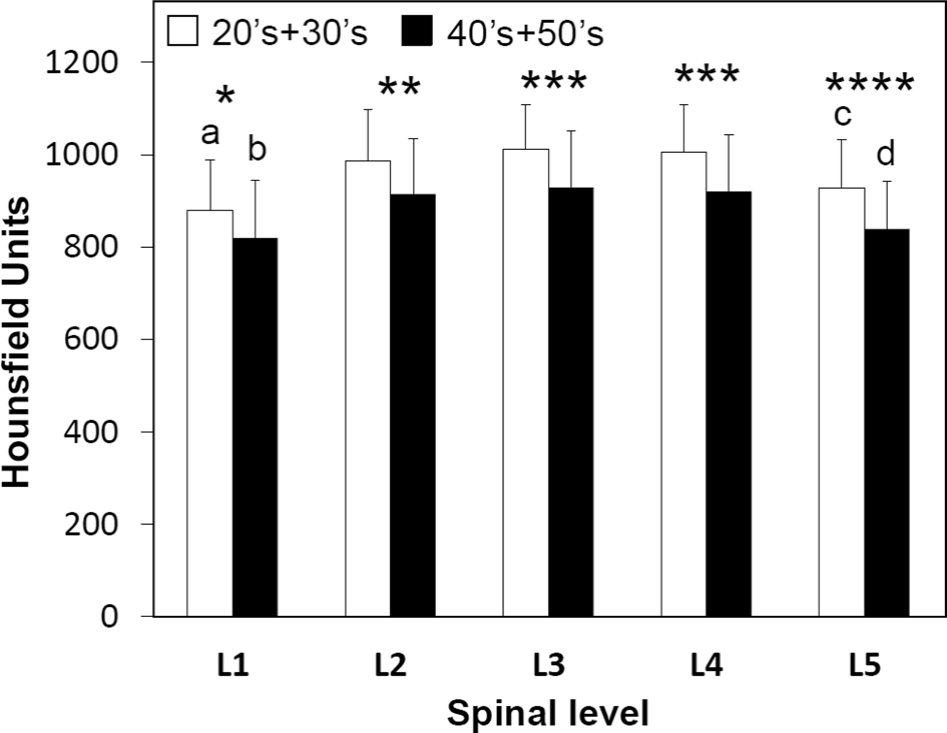
Attenuation split by spinal level and age. *p < 0.04 between age groups, **p < 0.009 between age groups, ***p < 0.002 between age groups, ****p < 0.0006 between age groups, (**a**) *p* < 0.0001 compared with L2, L3 and L4 and *p* < 0.03 compared with L5 in the same age group, (**b**) *p* < 0.0001 compared with L2, L3 and L4 in the same age group, (**c**) *p* < 0.003 compared with L2 and *p* < 0.0001 compared with L3 and L4 in the same age group, (**d**) *p* < 0.02 compared with L2 and *p* < 0.002 compared with L3 and L4 in the same age group.

**Figure 6:**
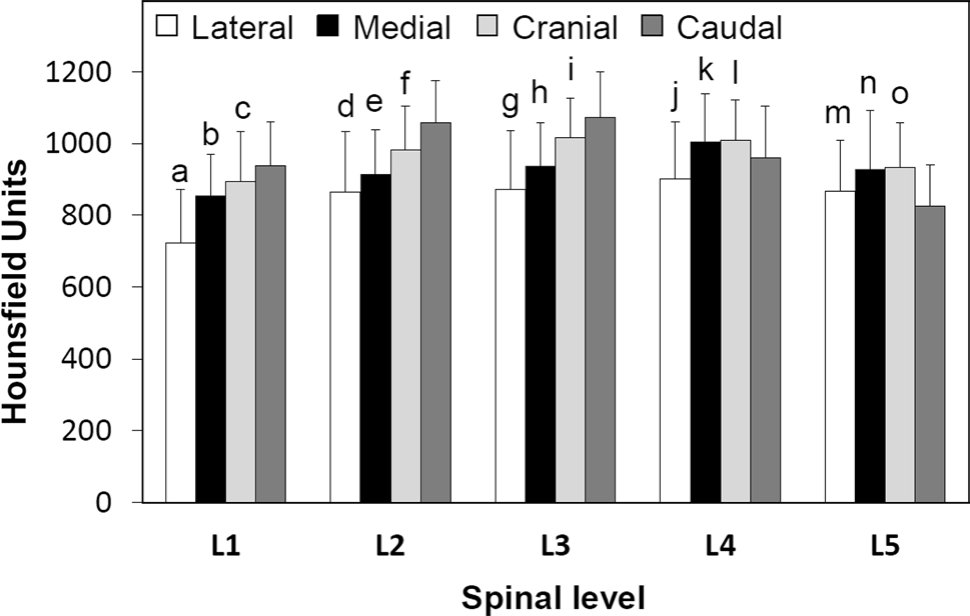
Attenuation split by spinal level and anatomical quadrant. (**a**) *p* < 0.0001 compared with medial, cranial and caudal in L1, (**b**) compared with cranial (*p* < 0.003) and caudal (*p* < 0.0001) in L1, (**c**) *p* < 0.0009 compared with caudal in L1, (**d**) compared with medial (*p* < 0.006), cranial (*p* < 0.0001) and caudal (*p* < 0.0001) in L2, (**e**) *p* < 0.0001 compared with cranial and caudal in L2, (**f**) *p* < 0.0001 compared with caudal in L2, (**g**) compared with medial (*p* < 0.0002), cranial (*p* < 0.0001) and caudal (*p* < 0.0001) in L3, (**h**) *p* < 0.0001 compared cranial and caudal in L3, (**i)** *p* < 0.0001 compared caudal in L3, (**j**) compared with medial (*p* < 0.0001), cranial (*p* < 0.0001) and caudal (*p* < 0.001) in L4, (**k**) compared with caudal (*p* < 0.05) in L4, (**l**) *p* < 0.003 compared with caudal in L4, (**m**) compared with medial (*p* < 0.007, cranial (*p* < 0.0001) and caudal (*p* < 0.004) in L5, (**n**) compared with caudal (*p* < 0.0001) in L5, (**o**) *p* < 0.0001 compared with caudal in L5.

The peak HU values in the pedicle wall were symmetric between both right and left sides. Considering spinal level, there were no significant differences by gender with the exception of L1 (*p* < 0.05) (Fig. 4, Table 3). The mean HU values in the 20 s/30 s group were higher than those in the 40 s/50 s (Fig. 5, Table 3). The mean HU values at L1 and L5 were significantly lower than the values between L2–4 in both genders and age groups (*p* < 0.03) (Figs. 4, 5, Table 3). From L1 to L3 the HU values followed this pattern: Lateral < Medial < Cranial < Caudal. The HU values in the lateral region were lower than those in the medial region at all spinal levels (*p* < 0.007) (Fig. 6, Table 3). The HU values at the caudal region were higher than those in the cranial region at L1, L2 and L3 (*p* < 0.0009, *p* < 0.0001 and *p* < 0.0001, respectively), however the HU values in the cranial region were higher than those in the caudal region at L4 and L5 (*p* < 0.003, *p* < 0.0001, respectively) (Fig. 6, Table 3). Pairing the pedicle wall HU and the vertebral HU by spinal level showed no correlation except at L4 in females and at L2 in males (Table 4). In the age group of 40’s + 50’s, these correlations were found at L2 and L4 (Table 4).

**Table 3:** Comparison of HU values by gender, age group and quadrant.

Gender, Age group and Quadrant	L1	L2	L3	L4	L5
Male	832.8 ± 100.0*^, a^	940.6 ± 103.3	964.9 ± 102.7	960.6 ± 96.8	865.5 ± 93.4^c^
Female	881.1 ± 126.2^b^	976.9 ± 127.7	991.6 ± 123.4	982.5 ± 134.7	914.3 ± 127.6^d^
20’s + 30’s	878.5 ± 109.4**^, e^	986.7 ± 110.0^†^	1011.7 ± 95.8^‡^	1006.0 ± 101.9^‡^	927.1 ± 106.0^§, g^
40’s + 50’s	827.4 ± 117.5^f^	921.5 ± 115.5	934.4 ± 120.0	926.6 ± 119.6	840.3 ± 103.9^h^
Lateral	726.2 ± 145.2^i^	865.8 ± 169.0^l^	875.9 ± 158.8^o^	904.0 ± 156.5^r^	869.4 ± 141.8^u^
Medial	851.9 ± 122.4^j^	913.8 ± 125.0^m^	934.5 ± 128.2^p^	1002.3 ± 139.6^s^	926.4 ± 164.8^v^
Cranial	894.5 ± 138.5^k^	981.5 ± 124.0^n^	1017.4 ± 110.7^q^	1008.3 ± 113.5^t^	932.7 ± 125.0^w^
Caudal	937.4 ± 124.0	1058.3 ± 117.3	1072.4 ± 127.1	958.9 ± 145.4	824.8 ± 115.8

*p < 0.05 between males and females.

**p < 0.04 between age groups.

^†^*p* < 0.009 between age groups.

^‡^*p* < 0.002 between age groups.

^§^*p* < 0.0006 between age groups.

^a^*p* < 0.0001 compared with L2, L3 and L4 in males.

^b^*p* < 0.0001 compared with L2, L3 and L4 in females.

^c^Compared with L2 (*p* < 0.0006), L3 (*p* < 0.0001) and L4 (*p* < 0.0001) in males.

^d^Compared with L2 (*p* < 0.05), L3 (*p* < 0.003) and L4 (*p* < 0.003) in females.

^e^*p* < 0.0001 compared with L2, L3 and L4 and *p* < 0.03 compared with L5 in the same age group.

^f^*p* < 0.0001 compared with L2, L3 and L4 in the same age group.

^g^*p* < 0.003 compared with L2 and *p* < 0.0001 compared with L3 and L4 in the same age group.

^h^*p* < 0.02 compared with L2 and *p* < 0.002 compared with L3 and L4 in the same age group.

^i^*p* < 0.0001 compared with medial, cranial and caudal in L1.

^j^Compared with cranial (*p* < 0.003) and caudal (*p* < 0.0001) in L1.

^k^*p* < 0.0009 compared with caudal in L1.

^l^Compared with medial (*p* < 0.006), cranial (*p* < 0.0001) and caudal (*p* < 0.0001) in L2.

^m^*p* < 0.0001 compared with cranial and caudal in L2.

^n^*p* < 0.0001 compared with caudal in L2.

^o^Compared with medial (*p* < 0.0002), cranial (*p* < 0.0001) and caudal (*p* < 0.0001) in L3.

^p^*p* < 0.0001 compared cranial and caudal in L3.

^q^*p* < 0.0001 compared caudal in L3.

^r^Compared with medial (*p* < 0.0001), cranial (*p* < 0.0001) and caudal (*p* < 0.001) in L4.

^s^Compared with caudal (*p* < 0.05) in L4.

^t^*p* < 0.003 compared with caudal in L4.

^u^Compared with medial (*p* < 0.007, cranial (*p* < 0.0001) and caudal (*p* < 0.004) in L5.

^v^Compared with caudal (*p* < 0.0001) in L5.

^w^*p* < 0.0001 compared with caudal in L5.

**Table 4:** Correlations between pedicle HU values and vertebral body HU values by gender and age group (coefficient and *p* value).

Parameter	HU values				
	L1	L2	L3	L4	L5
Male (n = 39)	0.23 (*p* = 0.15)	**0.45** **(p** <** 0.004)**	0.11 (*p* = 0.52)	0.26 (*p* = 0.11)	0.31 (*p* = 0.053)
Female (n = 36)	0.25 (*p* = 0.14)	0.14 (*p* = 0.42)	0.19 (*p* = 0.27)	**0.41** **(p** <** 0.02)**	0.01 (*p* = 0.95)
20’s + 30’s (n = 42)	0.23 (*p* = 0.14)	0.02 (*p* = 0.91)	0.03 (*p* = 0.83)	0.25 (*p* = 0.12)	0.12 (*p* = 0.43)
40’s + 50’s (n = 33)	0.22 (*p* = 0.22)	**0.52** **(p** <** 0.003)**	0.16 (*p* = 0.37)	**0.41** **(p** <** 0.02)**	0.12 (*p* = 0.50)

No correlation was found between pedicle HU values and vertebral body HU values at 20’s + 30’s at all levels. A positive correlation was found between pedicle HU values and vertebral body HU values in male L2 (R = 0.45, *p* < 0.004), female L4 (R = 0.41, *p* < 0.02), 40’s + 50’s L2 (R = 0.52, *p* < 0.003) and 40’s + 50’s L4 (R = 0.41, *p* < 0.02) shown in bold.

## Discussion

Although previous studies reported on the pedicle cortical bone thickness^7,19–23^ and the attenuation values of the *whole* pedicle or trabecular bone *inside* the pedicle^32–34^, the present study is the first to evaluate in vivo the regional attenuation distribution of the lumbar spine pedicle wall in all lumbar levels using clinical CT data. This report showed that there was an increase in HU values of the pedicle wall from L1 to L2, followed by a consistent plateau of data between L2 and L4 and a decrease at L5 in both gender groups and both age groups. Our regional distribution of attenuation showed that the lateral region had lower HU values than the medial region at all spinal levels. We also showed that HU values in the caudal region were higher than in the cranial region from L1 to L3, but lower at L4 and L5.

The increase seen in the average HU values from L1 to L2–4 may be attributed to a corresponding gradual rise in loading at the lower levels. However, the average HU values decreased between L4 and L5, which is consistent with results of previous research on the whole lumbar pedicle HU values, including the pedicle cortex and interior trabecular bone^32^. This finding may be explained by remarkable changes in pedicle dimensions between L4 and L5. Sugisaki et al*.* reported a significant increase in longitudinal axis of the pedicle isthmus from L4 to L5 by 25% and 27% in females and males, respectively^30^. Assuming pedicle cross-sectional geometry has an elliptical shape, these increases in the longitudinal axis would increase area moment of inertia to resist a bending moment in females and males by 95% and 105%, respectively. Therefore, the reduced HU values in L5 may be of limited influence on pedicular structural properties, leading to consider that a reduction of pedicular HU values in L5 may be a result of functional adaptation of bone tissue under reduced stress caused by the increased area moment of inertia.

The lateral region showed lower HU values than the medial region at all spinal levels. It has been reported that lateral wall breaches are the most common pedicle-screw related failures in thoracolumbar indications^12,20–22,35^ Previous studies looking at regional variation of the cortical thickness in the lumbar pedicle found that the cortical thickness was thinnest in the lateral region^7,12,21–23^, which has been thought to be a reason for the higher incidence of the pedicle penetration in the lateral pedicle wall. Crawford et al*.* investigated the trajectory of self-centering lumbar pedicle screws inserted in trajectories starting 0°, 10°, 20° or 30° from the optimal trajectory, either medially or laterally misdirected. The authors found that lateral misalignment as small as 10° was likely to lead to cortical wall violation (3 of 7 violations) whereas medial misalignment usually resulted in safe screw insertion (1 of 21 violations for 10°, 20°, or 30° medial misalignment)^12^. This study indicates that the lateral wall has less strength to resist pedicle screw penetration as compared with the medial wall. Although multiple factors would be involved in the higher incidence of pedicle screw breach in the lateral side, lower attenuation in the lateral pedicle wall shown in the present study should be considered as an important factor favoring pedicle screw breach due to the strong correlation shown by previous efforts between HU values and cortical bone material properties^36^.

Significant differences in the HU values between the cranial and caudal regions of the pedicle were found in the present study. While the HU values in the caudal region were higher at L1–3, the opposite case was seen at L4–5. This is clinically important in that the inferior breach of the pedicle screw at L4 and L5 is relatively common and can result in neurological complications. The surgeon should be mindful of the cortical walls with lower HU values, which are lateral in all lumbar levels and inferior at L4 and L5. Trabecular tracts running obliquely from the superior process downward to the inferior endplate through adjacent to the cranial cortex of the pedicle and from the inferior process upward to the superior endplate through adjacent to the caudal cortex of the pedicle were reported as early as 1925 by Gallois and Japoit^37^. The architecture of the trabecular bone tract trajectories connecting the articular processes and the lumbar vertebral body^38^ and radiographically dense trabecular tracts extending from the pedicles^15^ were also reported. Given that these trabecular tracts connecting the articular processes and the vertebral body bear load transmission between the anterior and posterior columns, the pedicle cortex in the cranial and caudal regions may also play a part in load transmission and resist bending moments borne by the pedicle. A full explanation of why are there higher HU values in the caudal region at L1–3 and in the cranial region at L4–5 remains elusive. We speculate that load transmission patterns between the anterior and posterior column through the pedicle, especially bending in the sagittal plane, may be different between the upper and lower lumbar spines, where spinal curvature and posture may play a role. Future biomechanical studies on the stress/strain distribution in the pedicle and their correlation with the HU values distribution will be required to explain the regional differences in pedicle wall HU values.

In the present study, we provided a new cylindrical HU dataset for each pedicle. HU values of new points in the cylindrical dataset were calculated by trilinear interpolation of the eight adjacent HUs in the original CT voxels (Fig. 1C). In the discrete cylindrical coordinate system, we used a smaller voxel size in the discrete cylindrical coordinate system than the original CT voxel size to minimize reduction of the peak HU values associated with trilinear interpolation. It should be noted that the sub-voxel position data of each point in the cylindrical coordinate system were not used for any geometrical analyses of the pedicle in the present study except for quadrant zoning of the pedicle.

Our study is not without some limitations. First, since this study cohort included subjects from 20 to 50 s, who are relatively active and still young, only two subjects met the criteria of osteoporosis defined by the vertebral HU values at L1^31^. Future studies including an osteoporotic population will be required*.* Second, we analyzed regional variations in pedicle wall attenuation by quadrant regions along the pedicle axis even though the entire 3D HU values distribution data were available. Analyses using other zoning systems and/or pattern analyses of the HU values distribution considering cortical bone trajectory would be of interest. Finally, the voxel size of the original CT data used in the present study, approximately 0.4 × 0.4 × 1.0 mm, may not be of high-enough resolution when compared with the pedicle cortical thickness if the intent was to evaluate absolute peak HU values at individual points due to partial volume effects.

In summary, this paper presented a novel 3D pedicle model based on peak HU values located radially away from the pedicle axis and investigated regional variation in lumbar pedicle wall attenuation. The results of present study demonstrated that the mean attenuation value at L5 was significantly lower than the values in L2–4 regardless of gender and age and the HU values in the lateral region was lower than in the medical region at all spinal levels. Since the 3D HU values distribution in the pedicle wall can be measured directly from patient-specific DICOM datasets without creating 3D models, the presented method could be used as a preoperative planning for transpedicular fixation using clinical CT.

## Data Availability

The datasets generated during and/or analyzed during the current study are available from the corresponding author on reasonable request.
